# Fluid Forces Enhance the Performance of an Aspirant Leader in Self-Organized Living Groups

**DOI:** 10.1371/journal.pone.0114687

**Published:** 2014-12-15

**Authors:** Alessandro De Rosis

**Affiliations:** Department of Agricultural Sciences, University of Bologna, Bologna, Italy; University of Catania, Italy

## Abstract

In this paper, the performance of an individual aiming at guiding a self-organized group is numerically investigated. A collective behavioural model is adopted, accounting for the mutual repulsion, attraction and orientation experienced by the individuals. Moreover, these represent a set of solid particles which are supposed to be immersed in a fictitious viscous fluid. In particular, the lattice Boltzmann and Immersed boundary methods are used to predict the fluid dynamics, whereas the effect of the hydrodynamic forces on particles is accounted for by solving the equation of the solid motion through the time discontinuous Galerkin scheme. Numerical simulations are carried out by involving the individuals in a dichotomous process. On the one hand, an aspirant leader (AL) additional individual is added to the system. AL is forced to move along a prescribed direction which intersects the group. On the other hand, these tend to depart from an obstacle represented by a rotating lamina which is placed in the fluid domain. A numerical campaign is carried out by varying the fluid viscosity and, as a consequence, the hydrodynamic field. Moreover, scenarios characterized by different values of the size of the group are investigated. In order to estimate the AL's performance, a proper parameter is introduced, depending on the number of individuals following AL. Present findings show that the sole collective behavioural equations are insufficient to predict the AL's performance, since the motion is drastically affected by the presence of the surrounding fluid. With respect to the existing literature, the proposed numerical model is enriched by accounting for the presence of the encompassing fluid, thus computing the hydrodynamic forces arising when the individuals move.

## Introduction

Social interactions involve the decision processes emerging in many animal groups, such as flocks of birds, schools of fishes or swarms of insects [1]–[5]. In particular, these organized groups routinely perform several decisions which are often remarkably crucial to enhance their survival chances. In fact, a particular kind of benefit is identified in predatory avoidance in schools of fishes [6]–[11], consisting of evasive manoeuvres confusing the attacking predator [12]. On the other hand, increasing attention has been devoted to the leader identification process [13]–[18]. A better understanding of such phenomenon can have a huge impact in a lot of applications. Among these, one of the most interesting is related to the design of a drone which can guide the real fishes towards a prescribed direction. For example, in [19] the response of fishes has been widely investigated by varying several morphological characteristics of the robot, showing that its size and color pattern strongly influence fish preferences. In [20], the interactions between two social fish species, zebrafish and mosquitofish, and a zebrafish-like robot have been discussed, showing that the robot tends to attract the zebrafishes, whereas the others are repulsed. Intriguing findings have been highlighted in [21], where it has been found that the biomimetic locomotion can alter fish preferences, since the tail beating attracts more the fishes towards the robot rather than when it is motionless. In [22], scenarios characterized by different beating frequencies have been dissected, showing that fish preferences are strongly influenced by the motion pattern. In [23], experiments in a water tunnel have been performed, elucidating the influence of both robot color and beating frequency on fish behaviour. These findings corroborate biological evidence that salient aspects of fish schooling benefit from the presence of group leaders [17], [18]. Several works [24]–[28] showed how the number of informed individuals affects the decision-making and leader identification processes for the uninformed ones. Moreover, these results can be used to design artificial leaders that can regulate collective behaviour. In order to perform effective and accurate predictions of the behaviour of a group of individuals, a lot of numerical models have been developed [29]–[34]. Such methods are based on metric [15], [35], [36] or topological [37]–[41] collective behavioural rules, which describe the mutual interaction in terms of reciprocal repulsion, attraction and orientation tendencies.

In this paper, beside the uniquely *social* approaches, the prediction of the leader identification and decision making processes emerging in a set of self-organized individuals is enriched by accounting for the presence of the encompassing fluid. Specifically, the lattice Boltzmann (LB) method is adopted to predict the fluid dynamics [42]–[44]. The LB method has been preferred to standard Navier-Stokes based solvers due to its algorithmical simplicity and computational efficiency, especially if force computations are involved in the numerical simulations, as in the present paper. In order to account for the presence of an immersed solid body (i.e. the individuals), the Immersed boundary (IB) method [45]–[47] is employed. The choice of the IB method over the well-consolidated interpolated bounce-back rule [48]–[51] is motivated by its superior properties in terms of numerical stability and by its high accuracy and convergence features, as recently demonstrated by the author [52]. Moreover, the combination of the LB and IB methods leads to the implementation of a quite general algorithm [53], which has been successfully used by the author in a lot of practical applications, including flapping wing dynamics [54], [55], shallow waters [56], multiphase flows [57], and even non-Newtonian fluid [58], among the others. Once fluid forces have been computed, the solid dynamics, i.e. the one involving the individuals, is computed through the time discontinuous Galerkin (TDG) scheme, which is known to possess high properties in terms of stability, convergence and accuracy [59], [60]. On the other hand, the collective behavioural model á la Couzin [15], [35] is adopted to compute the space-time evolution of the group. An additional aspirant leader (AL) individual is added to the group and it is forced to move towards a prescribed direction. During its motion, it meets the group and a certain portion of the individuals follows it. The AL's performance are assessed by proposing a proper parameter, as discussed in the following. Scenarios characterized by different values of the fluid viscosity are investigated. Moreover, the effect of the size of the group is dissected too. Numerical findings show that the purely behavioural modelling is insufficient to predict the AL's performance if it is immersed in a fluid, since the fluid dynamics plays a crucial role. It is worth to notice that the novelty of the present work with respect to the existing literature is represented by the insertion and the investigation of the dependence of the leader identification and decision making processes on the encompassing hydrodynamic field.

The rest of the paper is organized as follows. In the Methods section, the adopted numerical methods are recalled. In the Results and Discussion section, findings from a numerical campaign are presented and discussed. Finally, some conclusions are drawn in the Conclusion section.

## Methods

Here, the adopted numerical methods are briefly recalled and the proposed algorithm of computation is presented.

### Collective behavioural model

According to [15], the spatial position 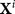 of the generic individual 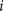 is updated in time 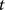 as follows
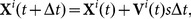
(1)where 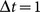 is the time step, 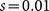 is the cruising speed and 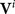 is the normalized velocity vector. Specifically, such quantity is computed as the summation of three contributions, i.e.
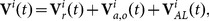
(2)where



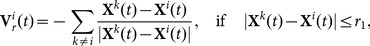
(3)

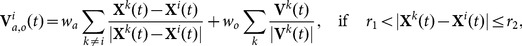
(4)

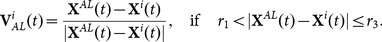
(5)


**Figure 1:**
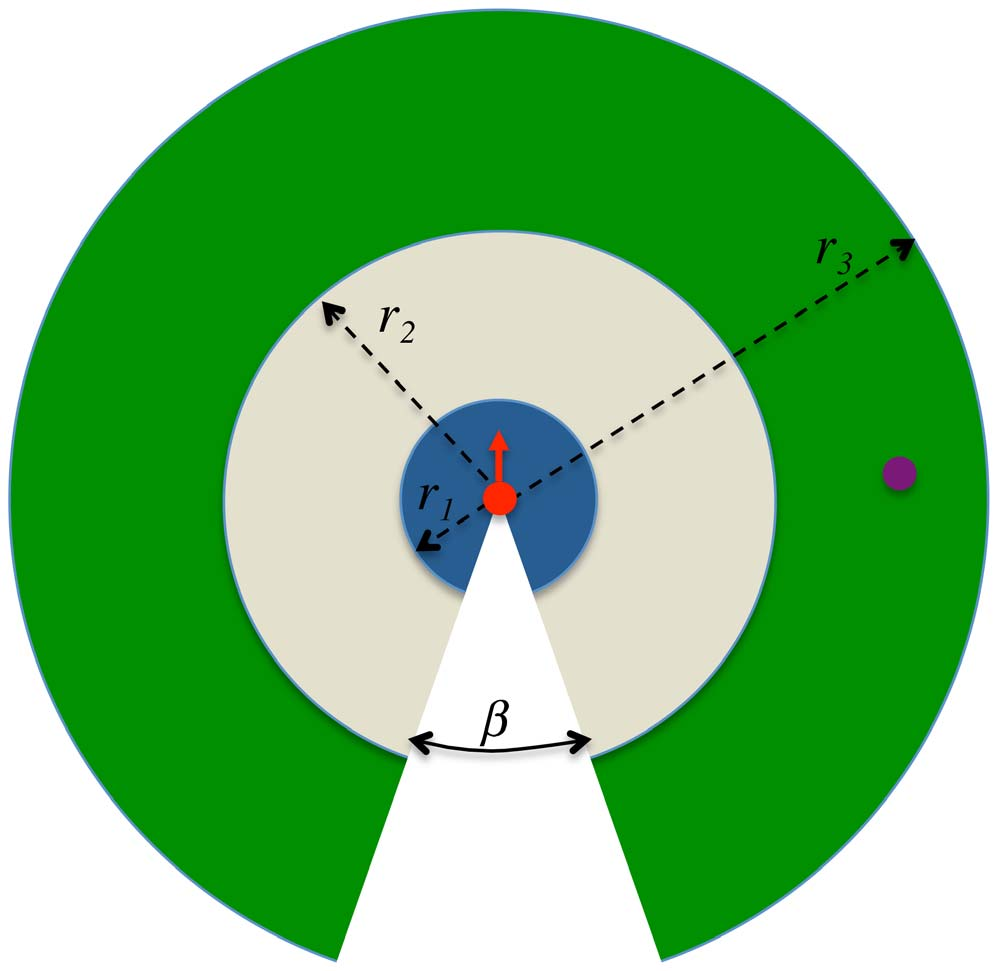
Schematic representation of the adopted behavioural model. The generic individual 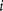 (red circle) moves along a direction (red arrow). Repulsion and attraction-orientation circular areas have radius 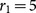 and 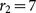, respectively. If AL (magenta circle) is detected in the area of radius 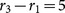, it exerts its influence on the individual 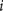. All the rules are neglected in the blind zone described by the angle 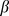.

Making reference to Fig. 1, the first equation accounts for the mutual repulsion and it is valid in a circular area of radius 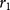. If the individual 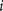 identifies another individual, namely 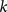, it moves in order to avoid collisions. The second one is the attraction-orientation equation: the individual 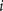 tends to get attracted and aligned with respect to individuals found in the area of radius 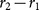. Attraction and orientation are weighted through proper coefficients, namely 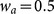 and 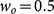, respectively. The third one is proposed by the author in order to account for the AL's influence. Specifically, AL is idealized as an additional individual which is located at the position 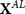. The generic individual 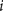 undergoes AL-induced attraction or orientation if the aspirant leader is detected in an area of radius 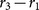. Notice that the area where the individual 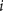 senses the AL's influence is intentionally set larger than the one of radius 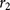, i.e. 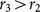. In fact, AL can be designed in order to be more persuasive than a real living individual, as discussed in the previous section. The individual 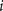 possesses a blind conic zone of angle 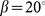 that is located in the direction opposite to the motion. Individuals which are detected here are completely neglected. Moreover, AL is forced to move towards the prescribed fixed point 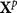. Therefore, the AL's velocity vector is computed as
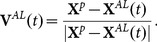
(6)


Concerning the presence of an obstacle (e.g. a rotating lamina), it is identified as an additional set of individuals with respect to the group is repulsed, whereas the attraction-orientation rule is neglected. Such tendency is enforced even for AL.

### The LB-IB-TDG strategy

**Figure 2:**
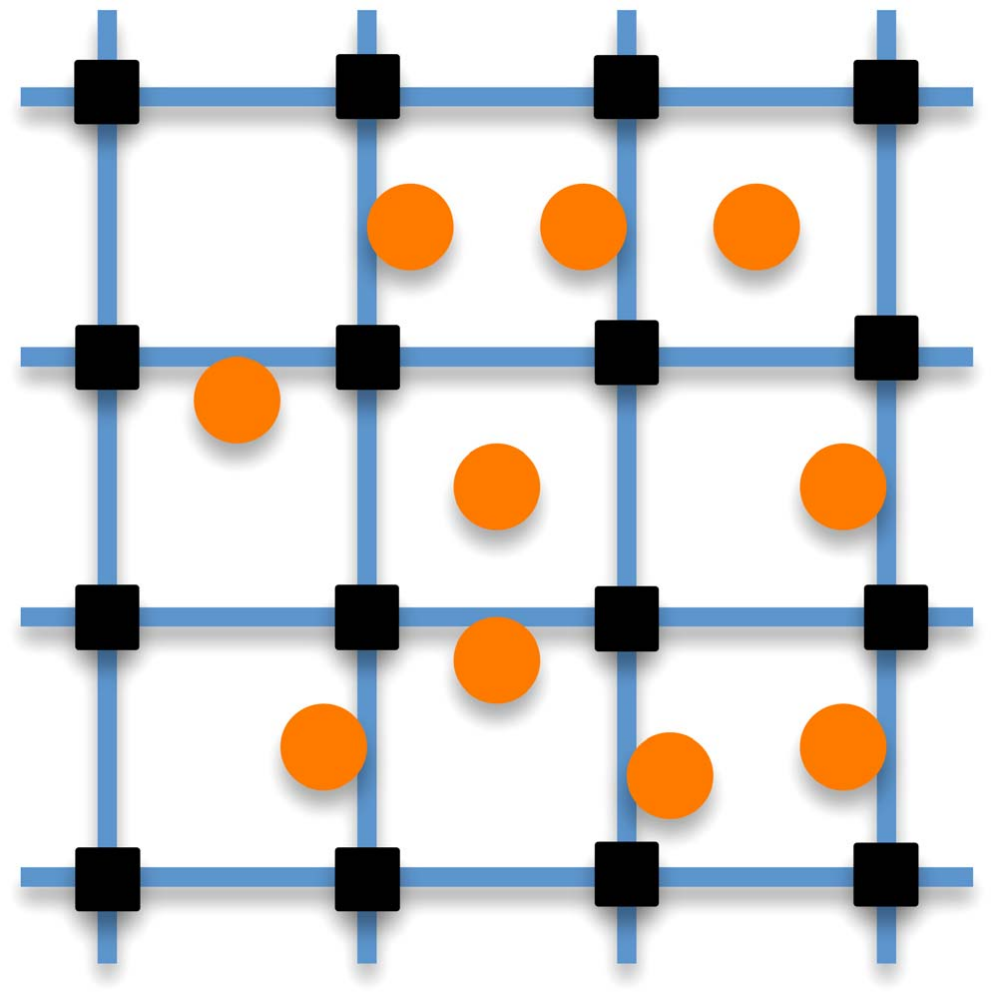
A set of particles (orange circles) are immersed in an Eulerian fixed square grid (blue lines). The black squares represent the lattice nodes.

The two-dimensional Bhatnagar-Gross-Krook [61] equation is solved on an Eulerian fixed square grid (see Fig. 2) where the particle distribution functions 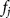 are forced to move along prescribed directions with velocities 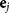 connecting the lattice nodes (black squares). This equation reads as follows
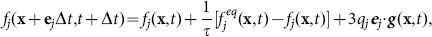
(7)where 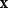 is the position, 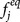 are the so-called equilibrium distribution functions [43], 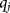 is a set of nine weights depending on the adopted LB D2Q9 model [44] and 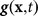 is a correction term. Given the relaxation time 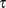, the fluid viscosity can be computed as 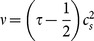, 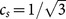 being the lattice sound speed, [44]. In the LB method, the macroscopic fluid density 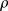 and the flow velocities 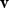 are easily computed as:
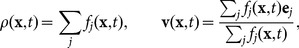
(8)respectively.

In order to account for the presence of an immersed body (orange circles) in the fluid lattice background at the position 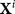, the Immersed Boundary method [45], [46] is adopted. The IB method has been implemented according to an implicit velocity-correction based procedure [47], [52], [53] which iterates in order to enforce the no-slip condition at the fluid-solid interface. A correction term 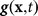 is calculated and it is used in the right-hand side of Equation 7. The computation of such term is highly important, since it leads rapidly to the force acting upon the immersed body as follows
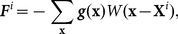
(9)

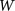
 being a kernel support [46]. Notice that the individuals and AL are idealized as circular objects of radius 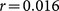.

In order to compute the time- and force-dependent displacements 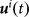 of an immersed body, i.e. the generic individual 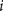, the equation of the rigid solid motion is solved, that is
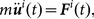
(10)where 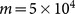 is the mass of the individuals, which is kept identical for the whole members of the system, and the time derivatives are indicated by superimposed dots. Equation 10 is numerically resolved by adopting the TDG algorithm. The interested reader can refer to [59] for further details about such time integration scheme. The author remarks that the strategy combining the LB, IB and TDG methods for solving fluid-structure interaction problems has already been widely validated [53]. In order to perform the conversion from lattice units to physical ones, the author selects a length scale factor 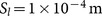, a velocity scale factor 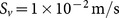 and a density scale factor 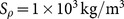.

### Algorithm of computation

**Figure 3:**
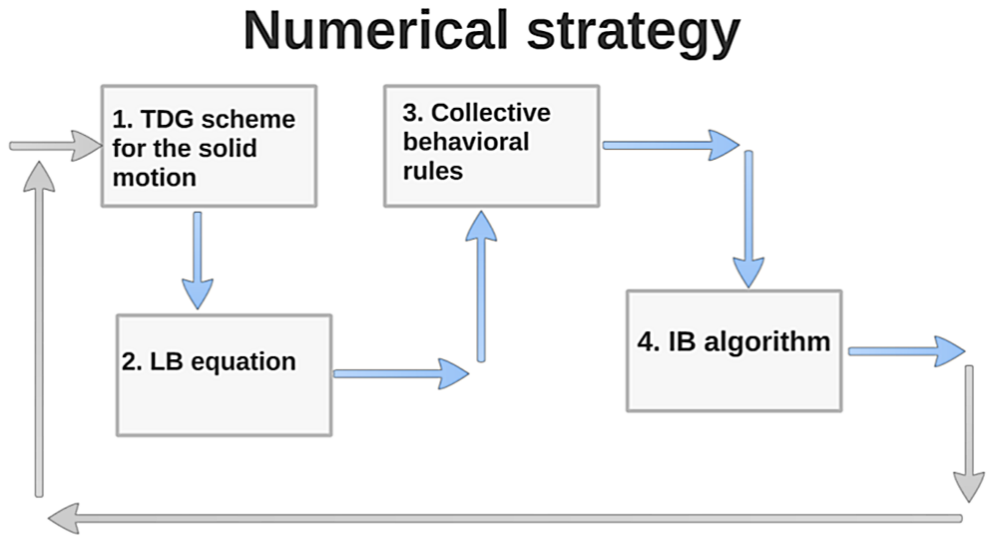
Sketch of the algorithm of computation.

The above mentioned numerical methods are combined in a proper strategy, consisting of an algorithmical sequence of actions which are sketched in Fig. 3. After assigning the initial values of the density and flow velocity, together with the initial position and velocity of the immersed bodies, the loop spanning the prescribed time range starts. In particular, the first action consists of solving the equation of the solid motion, i.e. Equation 10, for each individual. Thus, the positions and velocities of the individuals are updated. Notice that such action needs the displacements and velocities of the previous time step, together with the fluid forces. Secondly, the LB equation is solved by neglecting the last term in the right-hand side and the fluid macroscopic variables (i.e. 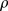 and 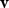) are computed. Thirdly, the position of the individuals is updated by solving Equation 1. Fourthly, the term 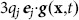 is computed through the implicit IB algorithm [52] and it is used to correct the particle distribution functions 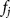 and the flow velocity 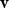. In order to advance in time, the algorithm repeats these tasks by using as initial conditions the displacements and velocities of the previous time step. The algorithm of computation is summarized in Table 1.

**Table 1:** Flow chart of the algorithm of computation.

0 - assign initial conditions and then start the loop on the time span;
1 - solve the equation of the solid motion for each individual,
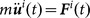 ;
2 - solve LB equation without the correction IB term,
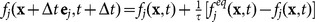 ;
3 - update the position of the individuals,
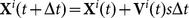 ;
4 - perform the IB implicit algorithm, correct 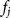 and 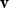 and advance in time going to step 1.

## Results and Discussion

**Figure 4:**
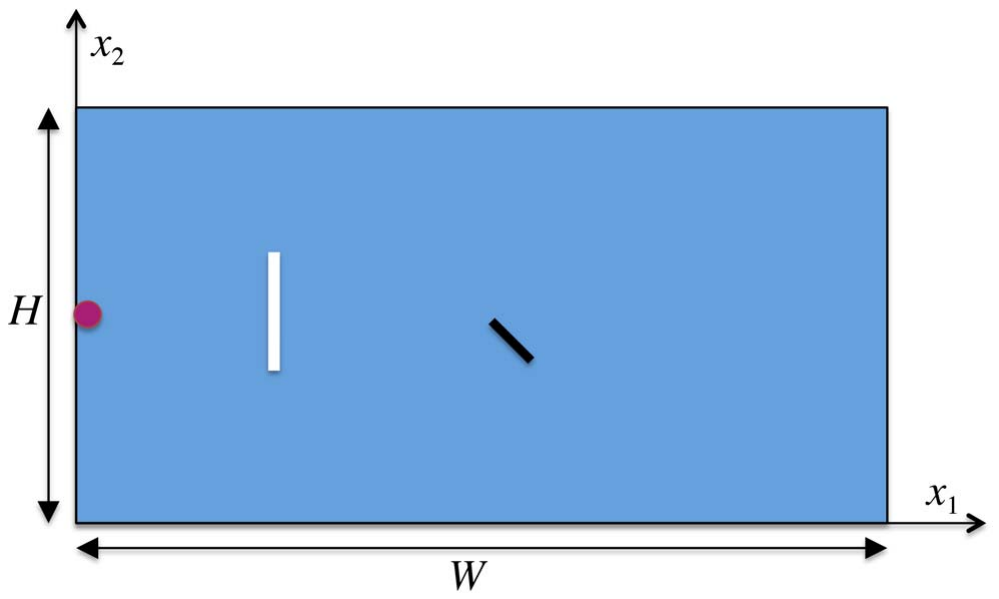
Sketch of the problem set-up. The individuals are represented by the white line. A lamina, whose top-most point is fixed at the center of the fluid domain, rotates harmonically. AL is placed at 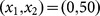 and it is enforced to move strictly rightward.

The problem set-up is sketched in Fig. 4. In particular, the fluid domain consists of 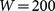 and 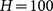 lattice nodes. AL is placed at 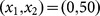. It is represented by the magenta point and it is forced to move towards the point 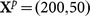. A lamina (black line), whose top-most point is fixed at the center of the fluid domain, undergoes a harmonic rotation, that is 
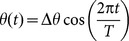
, where 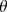 is the rotation angle, 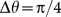 is the maximum angle and 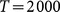 is the period of oscillation. The lamina is idealized by 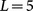 lattice nodes. The white line represents the initial position of the group. Notice that the top-most one is placed at 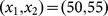 and the bottom-most one at 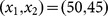. Depending on the number of individuals forming the group, the initial reciprocal spacing varies accordingly. All the simulations are characterized by a low Mach number, 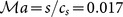, thus drastically annihilating deleterious compressibility effects which can affect the LB equation [44]. Scenarios characterized by progressively larger sizes of the group, 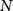, are investigated, i.e. 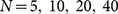. For a given value of 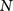, the effect of the fluid forces is elucidated by adopting different values of the fluid viscosity 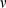, i.e. 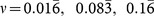. Findings are compared to a scenario characterized by the absence of the hydrodynamics. All the numerical simulations stop after 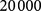 time steps.

**Figure 5:**
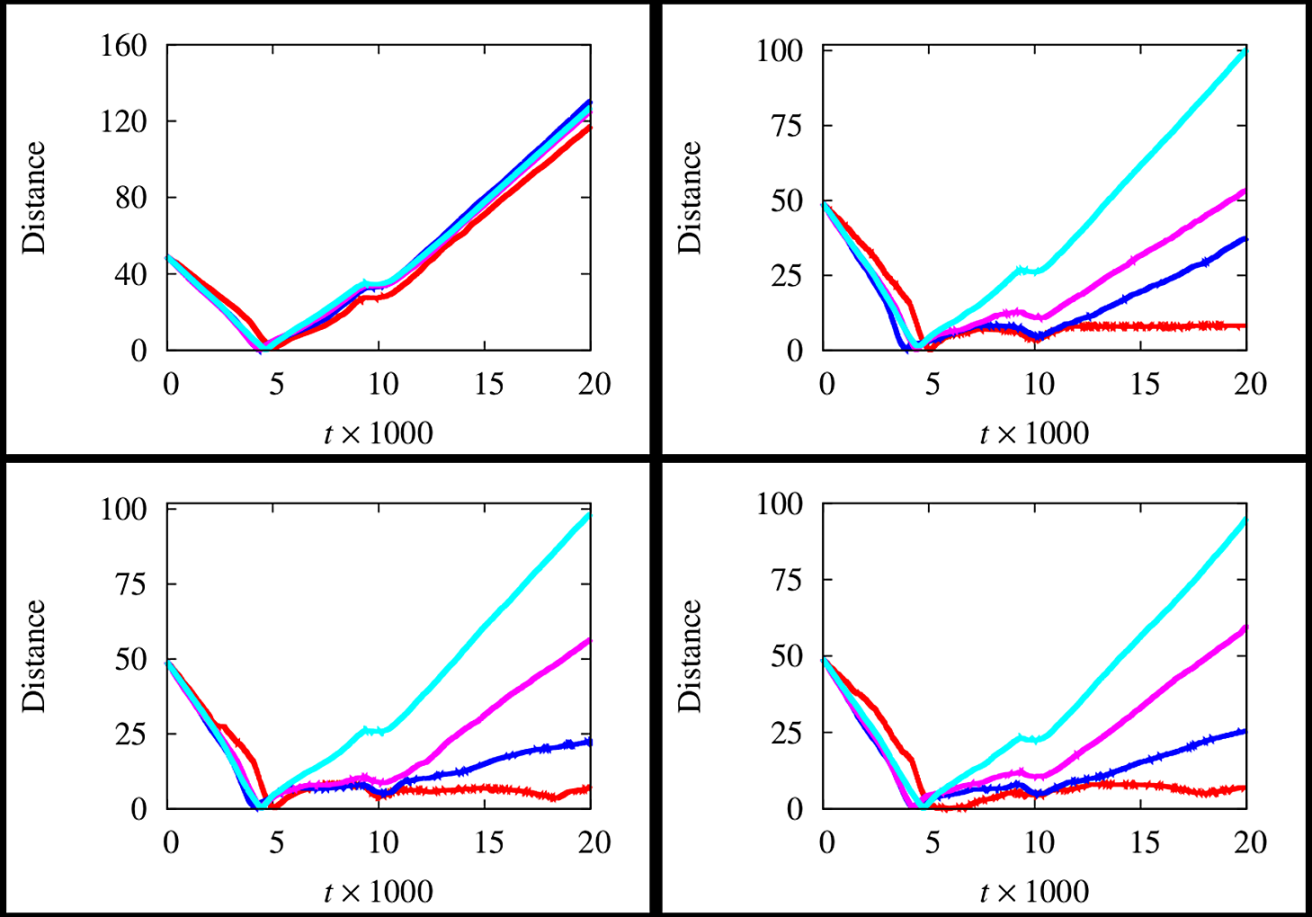
Time evolution of the distance between the group centroid and the position of AL for different values of 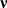, i.e. 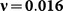 (top right panel), 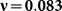 (bottom left panel), 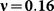 (bottom right panel), at 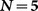 (red), 10 (blue), 20 (magenta), 40 (cyan). The scenario neglecting the fluid presence is reported in the top left panel.

**Figure 6:**
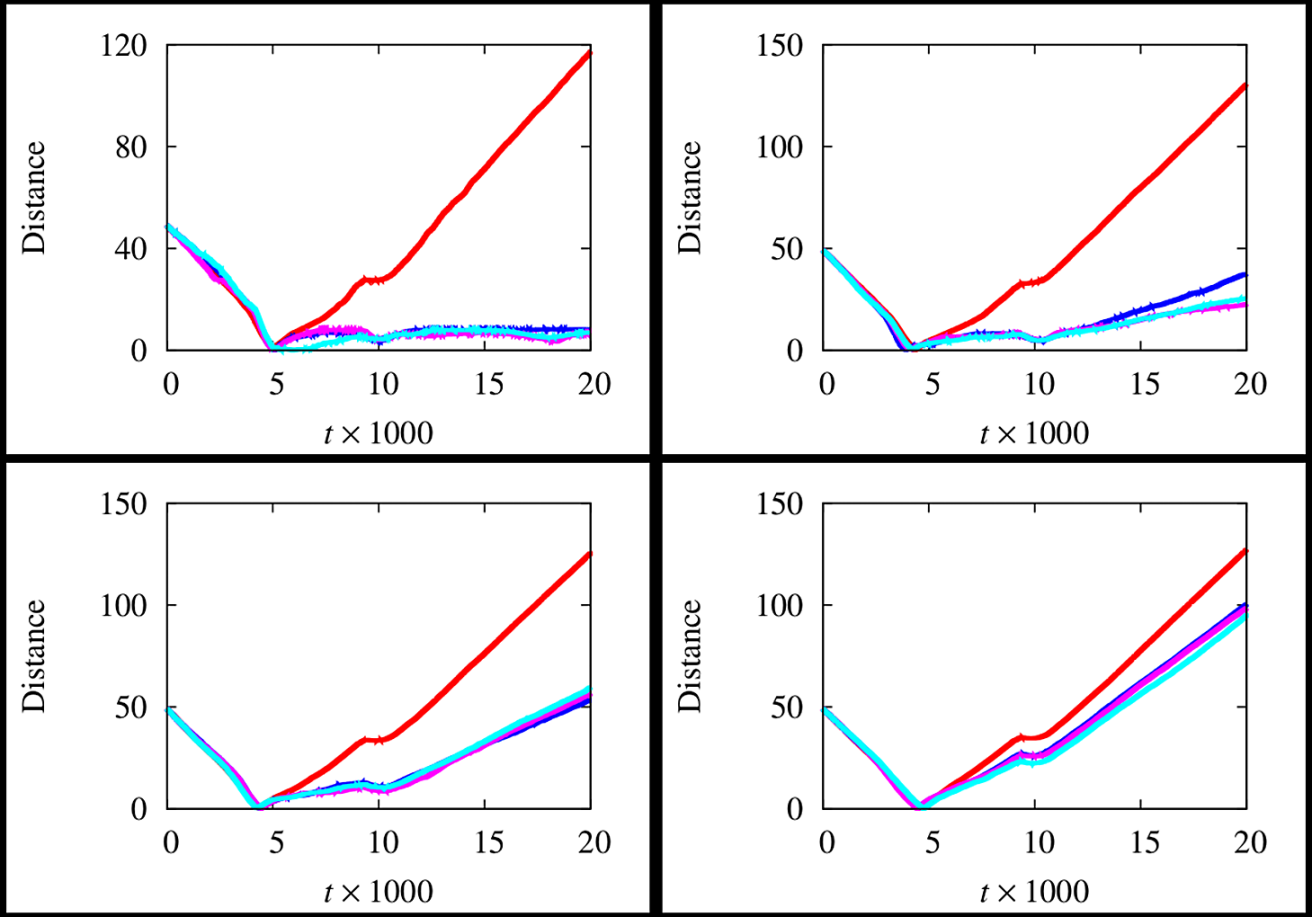
Time evolution of the distance between the group centroid and the position of AL for different values of 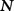. i.e. 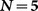 (top left panel), 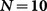 (top right panel), 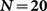 (bottom left panel), 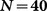 (bottom right panel), at 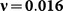 (blue), 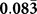 (magenta), 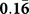 (cyan). **The scenario neglecting the fluid presence is denoted by the red curve.**

**Figure 7:**
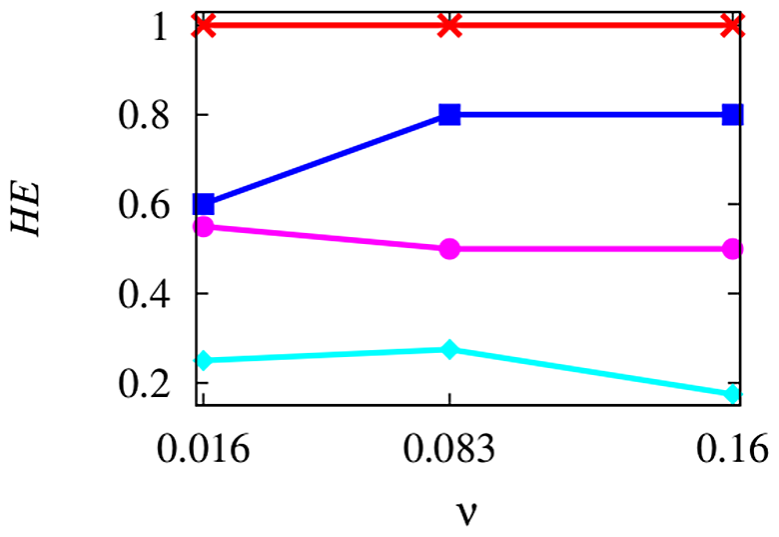
The hydrodynamic effect, 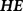, is reported for different values of 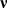, i.e. 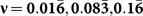, at 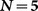 (red), 10 (blue), 20 (magenta), 40 (cyan).

In Fig. 5, the time evolution of the distance between the group centroid and the AL's position is depicted. Specifically, each graph refers to a value of 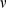 and four curves are reported, corresponding to the spectrum of the values of 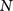. The scenario neglecting the fluid presence is reported too. As it is possible to observe, graphs show a common pattern until 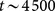, which represents the time employed by AL to reach the group. Therefore, the distance tends to reduce. In the remaining part of the considered time span, the scenario characterized by the absence of the hydrodynamics is drastically different with respect to the other ones. In particular, if the fluid is neglected the distance tends to grow in time, independently from the considered value of 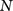. This means that AL passes through the group and it is unable to successfully attract the individuals. On the other hand, as 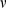 grows, such distance assumes lower values, thus allowing to assess that the group tends to get closer to AL. Therefore, the fluid presence plays a remarkably important role in the AL's performance. Such results are confirmed in Fig. 6, where each graph refers to a value of 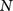 and four curves are reported, corresponding to the spectrum values of 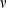. Specifically, it is worth to notice that the red curve, corresponding to the absence of the hydrodynamics, is considerably far from the curves representing the remaining configurations. Moreover, the effect of the size of the group is elucidated. In particular, notice that as 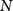 increases, the distance becomes larger. This means that, for a given value of 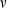, AL is more attractive as 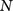 decreases. This suggests that AL is more effective for groups characterized by small sizes. The number of individuals following AL is reported in Table 2 for the all above mentioned scenarios. The author quantifies the improvement in the AL's performance by defining the hydrodynamic effect 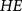, that is
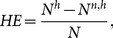
(11)where 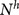 and 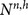 represent the number of individuals following AL in scenarios characterized by the presence and the absence of the hydrodynamics, respectively. In Fig. 7, the hydrodynamic effect is depicted. As above stated, the fluid-induced enhancement of the AL's performance is more marked as 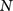 decreases.

**Table 2:** Number of individuals following AL for different values of 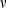 and 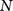.

	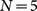	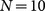	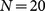	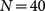
No fluid	0	0	0	0
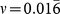	5	6	11	10
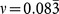	5	8	10	11
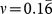	5	8	10	7

The scenario neglecting the fluid presence is reported too.

**Figure 8:**
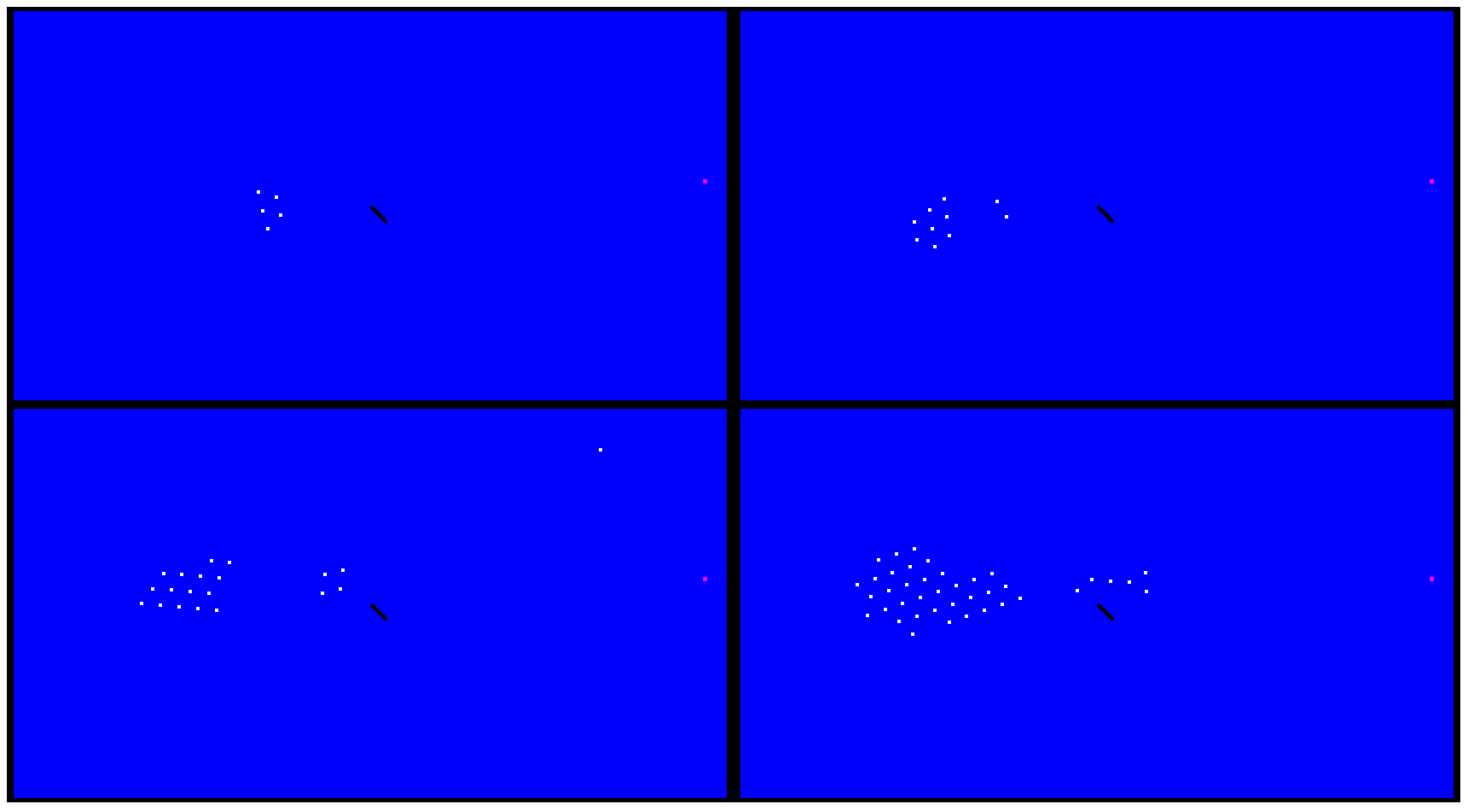
Absence of the hydrodynamics. Spatial configuration of the individuals (white) for different values of 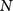, i.e. 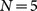 (top left panel), 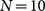 (top right panel), 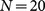 (bottom left panel), 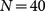 (bottom right panel). AL is coloured by magenta, while the rotating lamina is represented by the black solid line.

**Figure 9:**
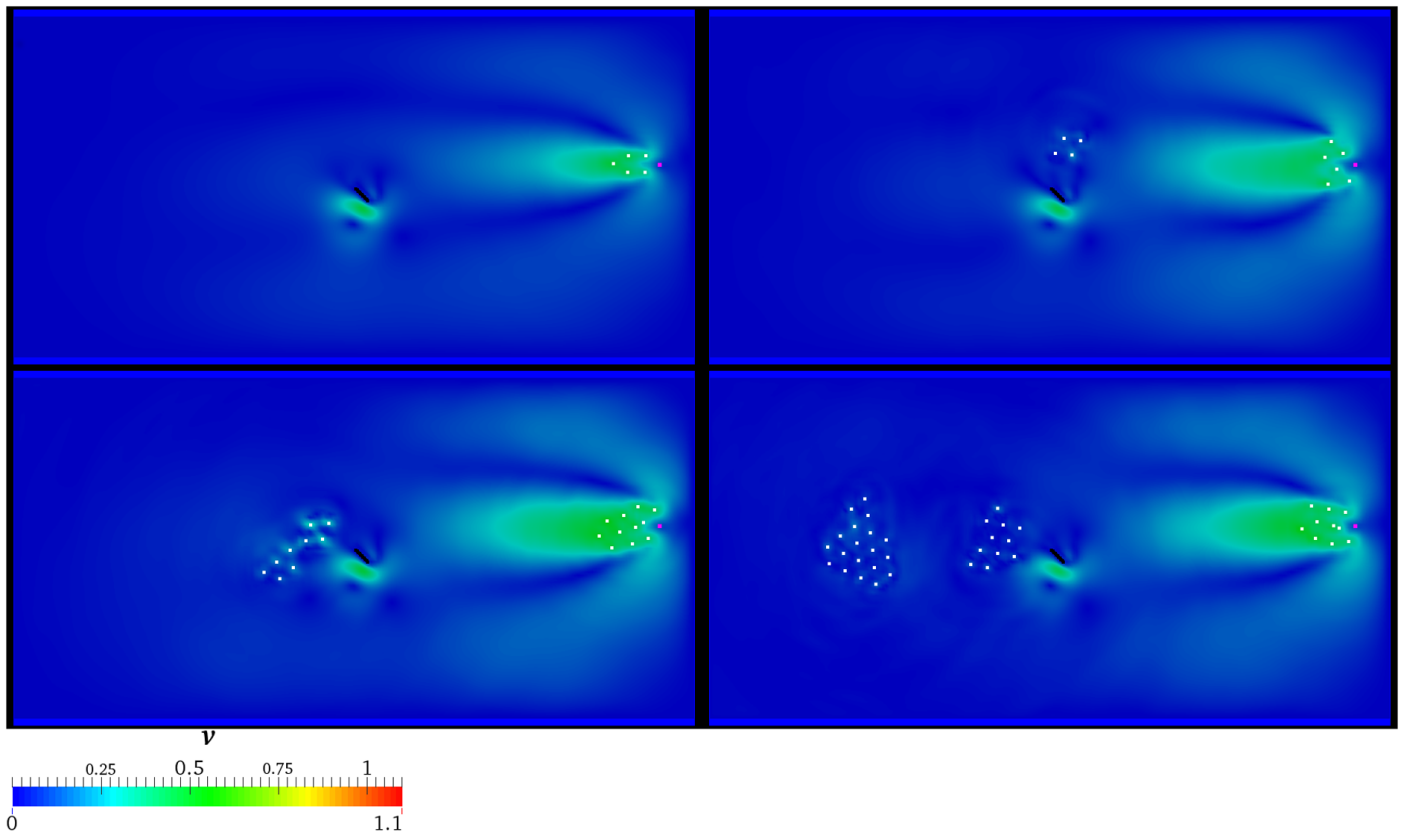
Presence of the hydrodynamics: 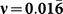. Velocity field and spatial configuration of the individuals (white) for different values of 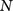, i.e. 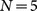 (top left panel), 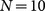 (top right panel), 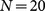 (bottom left panel), 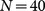 (bottom right panel). AL is coloured by magenta, while the rotating lamina is represented by the black solid line. The velocity magnitude is normalized by 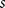.

**Figure 10:**
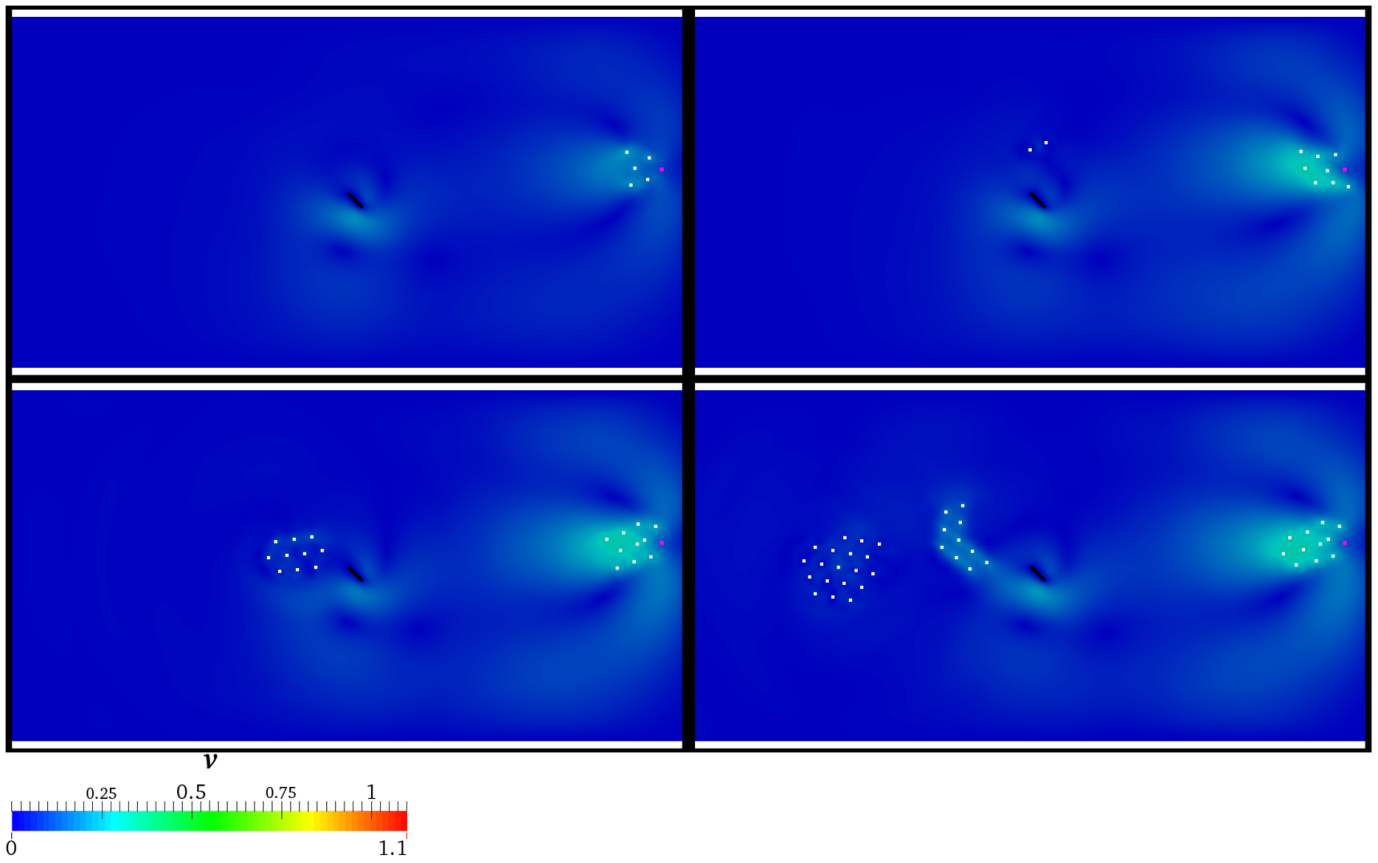
Presence of the hydrodynamics: 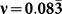. Velocity field and spatial configuration of the individuals (white) for different values of 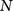, i.e. 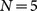 (top left panel), 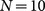 (top right panel), 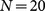 (bottom left panel), 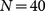 (bottom right panel). AL is coloured by magenta, while the rotating lamina is represented by the black solid line. The velocity magnitude is normalized by 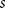.

**Figure 11:**
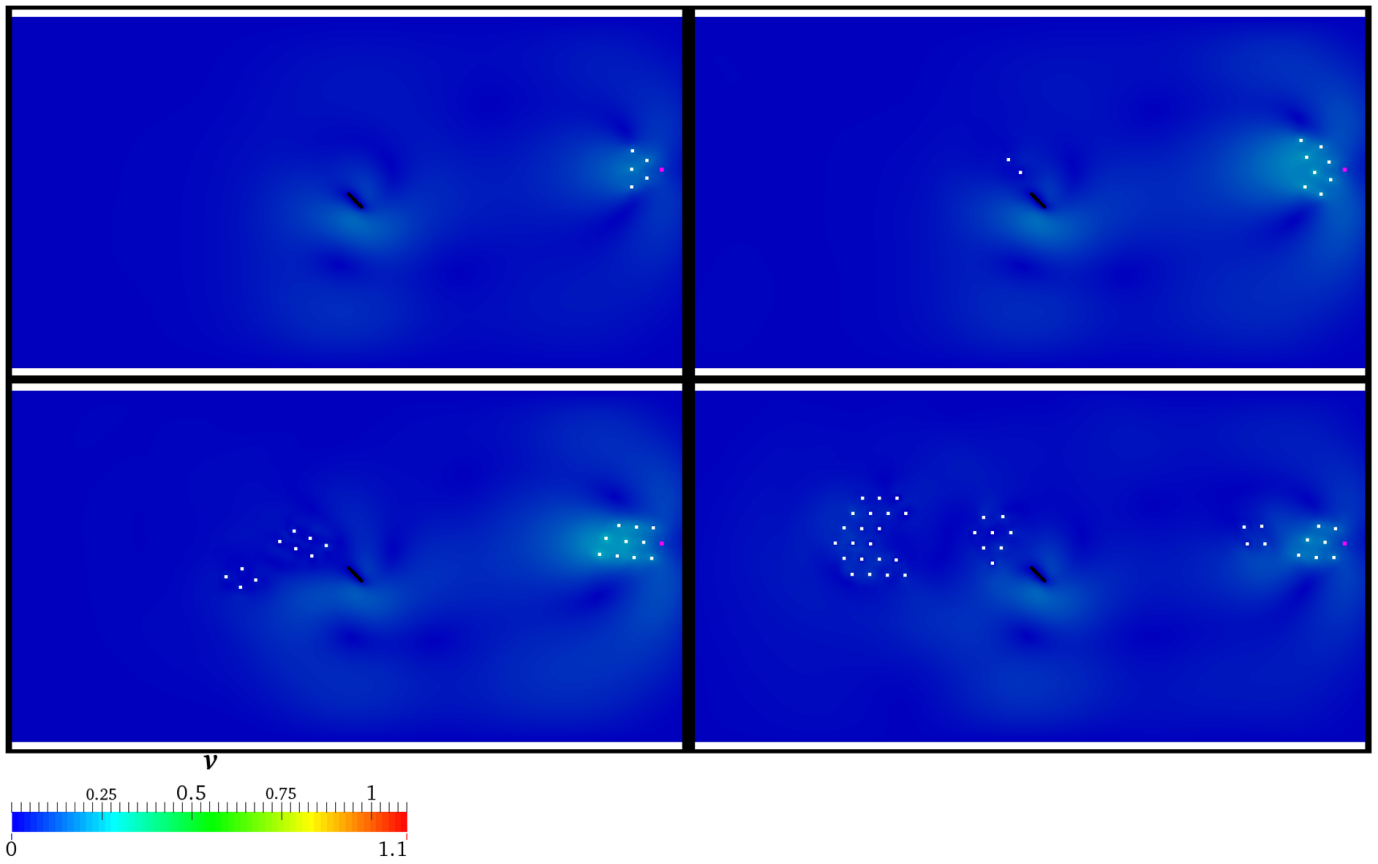
Presence of the hydrodynamics: 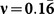. Velocity field and spatial configuration of the individuals (white) for different values of 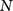, i.e. 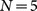 (top left panel), 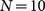 (top right panel), 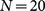 (bottom left panel), 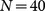 (bottom right panel). AL is coloured by magenta, while the rotating lamina is represented by the black solid line. The velocity magnitude is normalized by 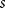.

**Figure 12:**
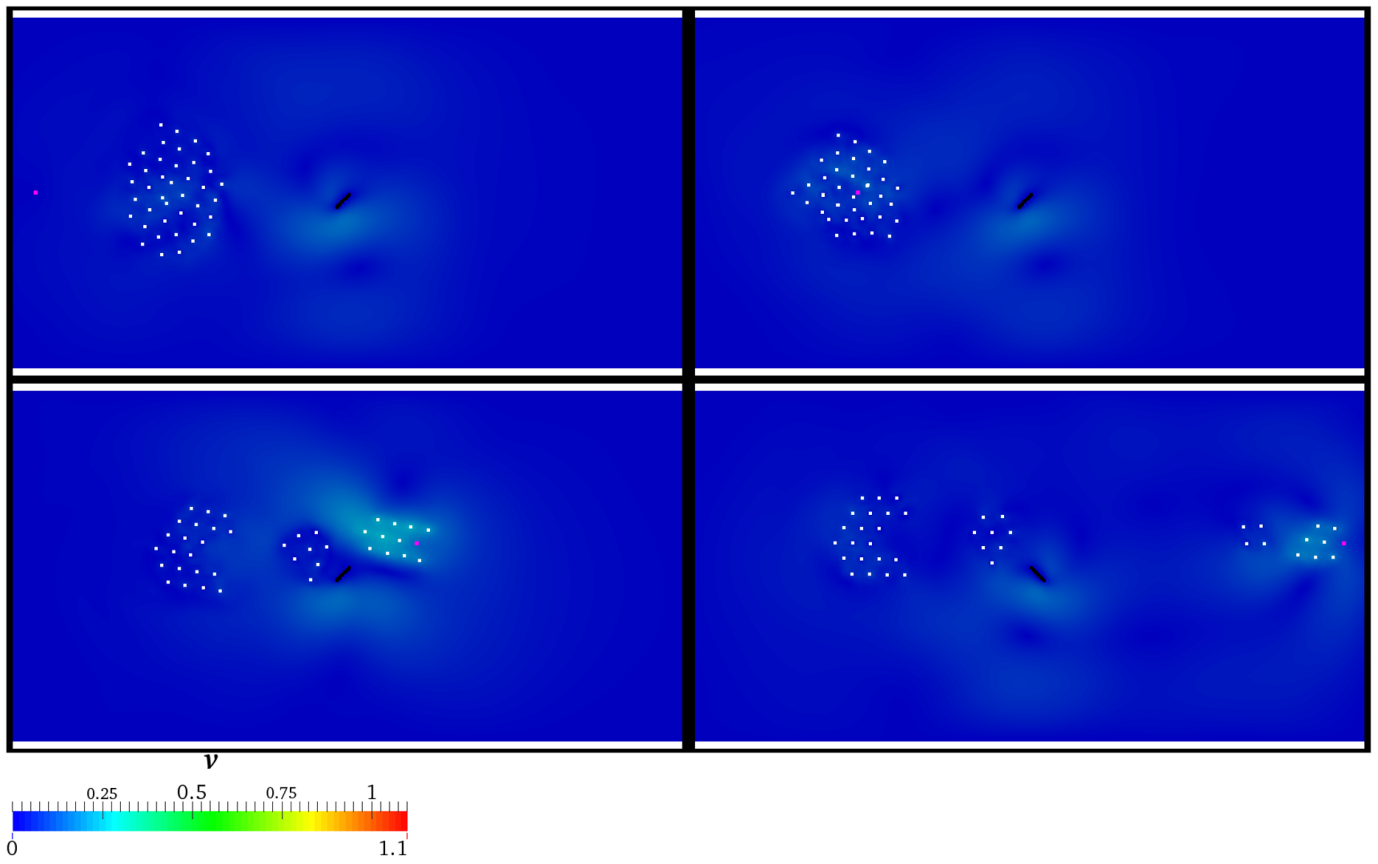
Velocity field and spatial configuration of the individuals (white) at different time instants, i.e. 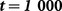 (top left panel), 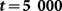 (top right panel), 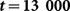 (bottom left panel), 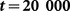 (bottom right panel), in the scenario characterized by 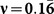 and 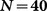. AL is coloured by magenta, while the rotating lamina is represented by the black solid line. The velocity magnitude is normalized by 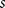.

A deeper investigation on the space-time evolution of the group is carried out by sketching the spatial configuration of the individuals at 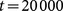 for the above discussed scenarios, thus highlighting the fragmentation of the group. Notice that it is induced by both AL and the lamina. In particular, in Fig. 8 scenarios characterized by the absence of the hydrodynamics are reported. As it is possible to observe, the group is progressively more elongated as 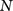 grows, due to the AL's transit. In particular, notice that at 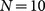 two individuals separate from the group. At 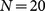, four individuals arrive close to the lamina, whose presence stops these. In this configuration, it is intriguing to notice that an individual tends to follow AL, but when it reaches the lamina, it is obstructed. Then, it loses the route, thus moving in an a priori unpredictable direction. At 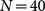, six individuals stall close to the lamina, whereas the remaining part falls behind, showing a certain level of elongation. Scenarios characterized by 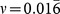 are depicted in Fig. 9. At 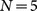, AL successfully attracts and guides the whole group. Such results are partially achieved at 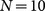, where four individuals stop their motion close to the lamina. At 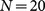, nine individuals miss the goal. Notice that two subgroups can be identified within these nine individuals: the former is represented by the four right-most ones which are obstructed by the lamina, the latter is composed by the remaining ones, which fall behind and then these tend to merge. At 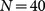, ten individuals are guided, eleven stall close to the lamina and the remaining nineteen are substantially torpid with respect to the AL's transit. If 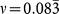 is considered (see Fig. 10), results discussed for the previous scenario are substantially confirmed, even if AL appears to be slightly more persuasive. Finally, it is interesting to notice that at 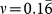 the splitting effect is more marked. Specifically, at 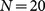 and 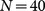 three and four well distinct subgroups arise, respectively, as reported in Fig. 11. Notice that the contour plot represents the velocity field. The color legend range is kept fixed in order to highlight the reciprocal difference among the various scenarios. In particular, the higher 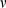, the lower the velocity magnitude is. For sake of completeness, the evolution of the velocity field is depicted in Fig. 12, together with the motion of the immersed individuals, in the scenario characterized by 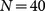 and 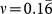 at three time instants, i.e. 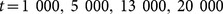.

Finally, notice that the author creates a website where the animations showing the velocity field and spatial configuration of the individuals are present (https://www.youtube.com/playlist?list=PL-z4SoMtQxJSHsrIKoZDimokoKf6klFFJ).

### The effect of a noise

**Figure 13:**
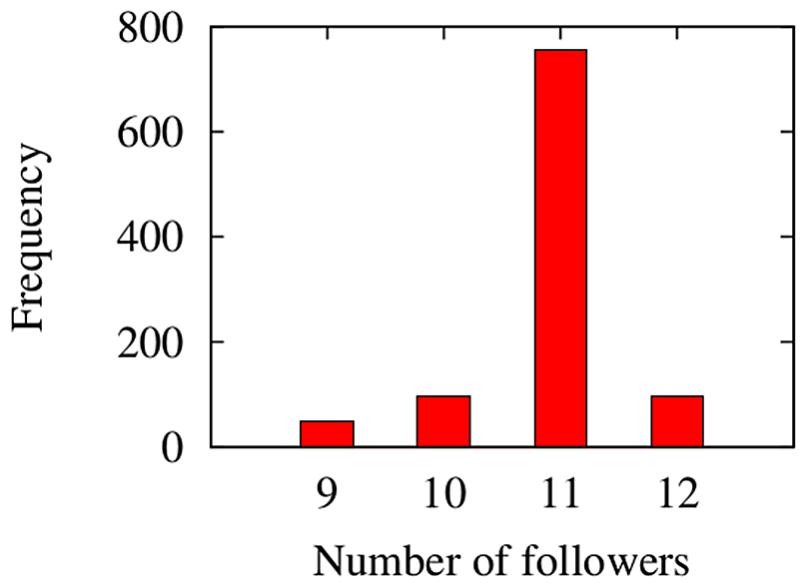
Frequency histogram of the number of individuals following AL at 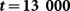. A mean value 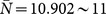 and a variance 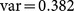 are achieved.

Previous simulations have been carried out from a deterministic point of view, by neglecting the presence of disorder and disturbance. As known, the dynamics of a group of self-organized individuals can be affected by several random conditions, such as the initial position or the orientation of the individuals. Here, the scenario characterized by 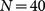 and 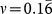 is employed to dissect the role of a noise. Specifically, at each time step the velocity vector of each individual is rotated by an angle 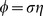 where 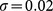 and 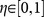 is a random number generated by a stochastic process. The author selects the number of individuals following AL at 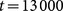 as a variable to be monitored. A Monte Carlo investigation is performed, consisting of 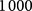 simulations. In Fig. 13, the frequency histogram of the monitored quantity is depicted. As it is possible to observe, about the 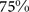 of the simulations leads to a number of followers equal to 11. On the other hand, the remaining part of the simulations affirms that the number of individuals following AL can be 9, 10 or 12, thus achieving a slight mismatch with respect to the more frequent result. In fact, a mean value 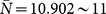 and a variance 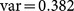 are achieved.

## Conclusions

In this paper, the collective dynamics of a group of self-organized individuals immersed in a fictitious viscous fluid has been investigated. The group interacts with an additional individual, AL, which aims at becoming a leader. The effect of the fluid forces has been elucidated by combining the LB-IB-TDG strategy for fluid and solid dynamics to the collective behavioural equations. It has been found that the fluid tends to create a more compact group, thus enforcing a deeper exchange of information among the individuals. As a consequence, AL can successfully guide the individuals. In addition, groups characterized by progressively larger size have been involved in the computations, showing a splitting effect which increases with 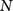. Present findings lead to assess that the behaviour of the group is considerably affected by the presence of the encompassing fluid. Therefore, an accurate computation of the fluid dynamics is crucial to successfully predict the behaviour of a multi-agent system immersed in a fluid. Notice that the proposed approach represents a substantial improvement with respect to a previous effort carried out by the author [62], where the effects of the hydrodynamics have been investigated for a predator-preys system by using a simple expression for the hydrodynamic force and by neglecting the mass of the individuals. Given the net effect exerted by the fluid, one could conclude that the fluid incidence could be easily incorporate in the purely behavioural equations by increasing the weight of the attraction rule, 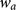, thus avoiding the computation of the fluid dynamics. Such approach can lead to wrong predictions about the collective motion. In fact, the fluid dynamics exhibits a strong non-linear dependence on 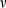 and the position of the individuals. According to [63], the members which are located in the rear part of the group can benefit from the vortexes characterized by particular wakes created by the front ones, since these experience reduced drag forces. Therefore, an accurate computation of the fluid dynamics represents an important aspect to be properly investigated, especially for fish-based studies. Further investigations will dissect the effect of the size of the wake. Specifically, the individuals will be modelled as deformable solid bodies [64], [65] moving in a fluid, aiming at elucidating the incidence of the vorticity field both on the single and collective motions.
